# *In vivo* biodistribution analysis of transmission competent and defective RNA virus-based episomal vector

**DOI:** 10.1038/s41598-020-62630-7

**Published:** 2020-04-03

**Authors:** Yumiko Komatsu, Chiaki Tanaka, Ryo Komorizono, Keizo Tomonaga

**Affiliations:** 1 Laboratory of RNA Viruses, Department of Virus Research, Institute for Frontier Life and Medical Sciences (inFront), Kyoto University, Kyoto, Japan; 2 Keihanshin Consortium for Fostering the Next Generation of Global Leaders in Research, Kyoto University, Kyoto, Japan; 3 Department of Mammalian Regulatory Network, Graduate School of Biostudies, Kyoto University, Kyoto, Japan; 4 Department of Molecular Virology, Graduate School of Medicine, Kyoto University, Kyoto, Japan

**Keywords:** Gene delivery, Viral vectors

## Abstract

RNA virus-based episomal vector (REVec) is an emerging viral vector system that mediates long-term stable gene expression in variety of cell types *in vitro*. However, little is known about its tissue tropism and persistence of gene expression *in vivo*. Here, to evaluate the feasibility of REVec for *in vivo* gene delivery, we conducted biodistribution analysis of transmission competent REVec and transmission defective ΔG-REVec in Lewis rats. Following intracranial administration of REVec, transgene expression was detected in various tissues. In contrast, transgene expression was only observed in the brain after ΔG-REVec administration. Low levels of vector shedding in the feces and blood and of neutralizing antibody in the serum were detected after REVec injection. In the brain, microglia, astrocytes and neurons were susceptible to REVec-mediated transduction. However, the animals administered with REVec, but not with ΔG-REVec showed a significant decrease in body weight compared to mock treated animals. Additionally, CD8 T cell infiltration was observed in the brain of these animals. In summary, we demonstrated that REVec promotes long-term transgene expression *in vivo* without causing high vector shedding or neutralizing antibody production; however, suggests the need to attenuate vector associated pathogenicity in the future.

## Introduction

Advances in gene delivery technologies to mammalian cells have greatly accelerated the development of gene therapy for treatment of human diseases. In particular, viral vector-based gene therapies have shown clinical benefit in patients with genetic diseases due to their high efficiency of gene transfer^1^. *In vivo* gene therapy involves direct injection of therapeutic gene in target tissues, thus eliminating the need for cell handling facilities, which are required in *ex vivo* gene therapy that involves isolation and modification of cells in culture. Among various viral vector technologies, adeno associated virus (AAV) vector has provided an excellent example of applicability of viral vectors for treatment of a genetic conditions by direct gene transfer approach^2–7^. In order to achieve therapeutic efficacy, *in vivo* gene transfer system that demonstrates sustained transgene expression is required for the patients to benefit from long-term therapeutic effect.

Borna disease virus (BoDV) is a neurotropic virus that causes persistent infection in many vertebrate species^8^. One of the most remarkable characteristics of BoDV is its ability to persist in the nucleus without the need for integration whereby viral ribonucleoprotein (RNP) binds and segregates with host chromosomes during cell division^9^. Additionally, BoDV can establish persistent infection without causing cytopathic effects^10^. These features of BoDV were previously exploited to develop an RNA virus-based episomal vector (REVec) as an non-integrating stable gene expression system^11^.

The first generation replication competent REVec that expresses an extra transcription unit between the phosphoprotein (P) and matrix (M) genes was initially established, followed by development of the second generation transmission defective vector which lacks an envelope glycoprotein (G) gene, ΔG-REVec^11^. More recently, the third generation vector which lacks both G and M genes, ΔMG-REVec was developed, and has shown persistent transgene expression in Vero cells^12^.

Both transmission competent REVec and defective ΔG-REVec have been successfully used to transduce different human-derived cell types *in vitro*, including oligodendrocytes^11^, glioblastoma derived cells^13^, medulloblastoma cells^14^, pancreatic embryonic kidney cells^11^, mesenchymal stem cells^13^ and induced pluripotent stem cells (iPSCs)^13,15^. In particular, we have recently reported that ΔG-REVec achieves highly efficient gene transfer to human iPSCs^15^, indicating its potential for *ex vivo* applications. Additionally, REVec with a pri-miRNA cassette have been established to knockdown a target gene by stable expression of miRNA^16^, further demonstrating its potential use in RNAi therapies.

Although REVec achieves long-term transgene expression in variety of cell types *in vitro*, its tissue tropism and efficiency of *in vivo* gene transfer has not been examined to date. To assess feasibility of REVec for direct *in vivo* gene delivery, in current study, we analyzed tissue tropism, vector shedding, and conducted serum analysis of transmission competent REVec and defective ΔG-REVec.

## Results

### *In vivo* imaging of REVec after intranasal and intracranial administration

**Figure 1:**
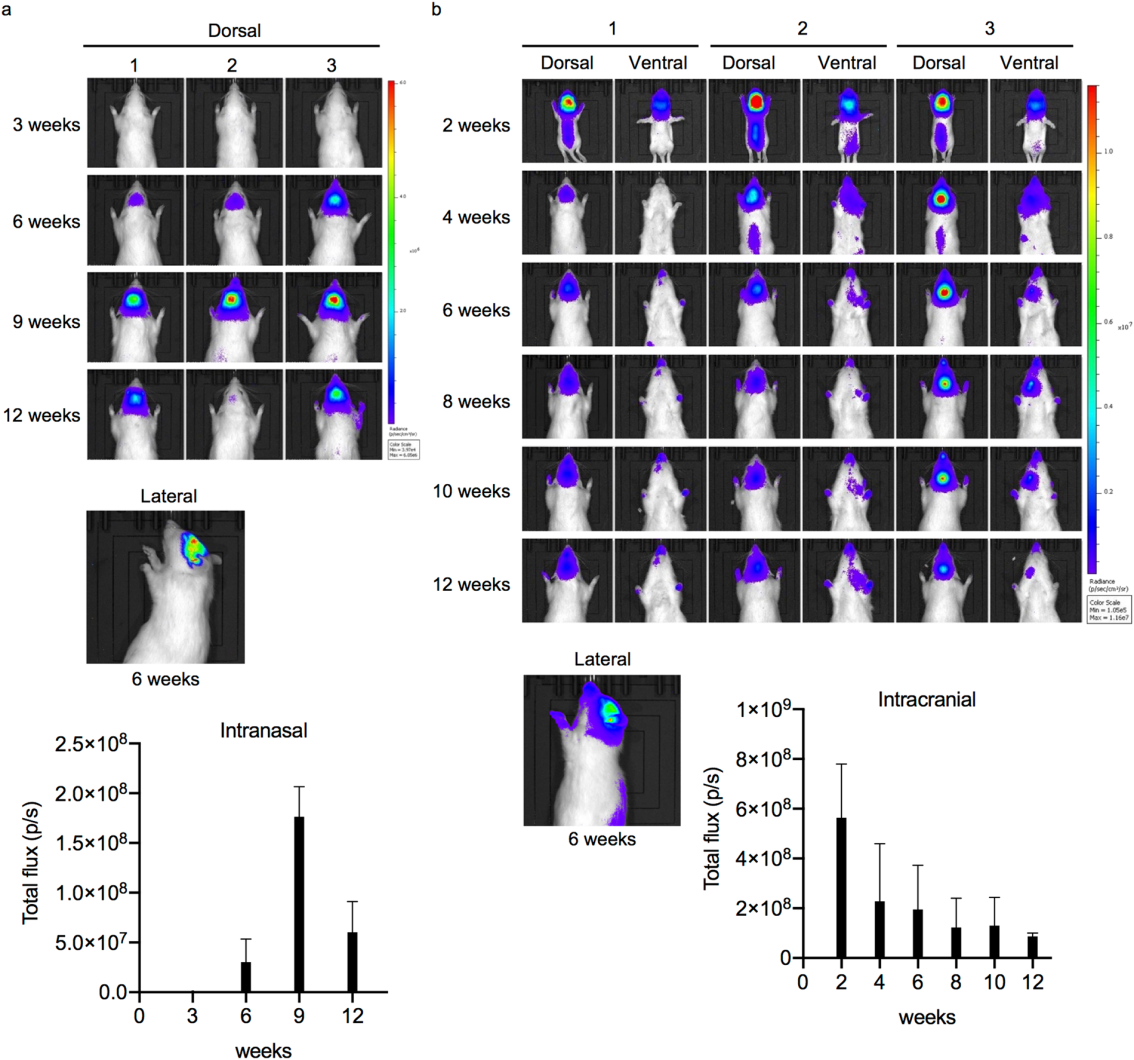
*In vivo* imaging of REVec. (**a**) *In vivo* luciferase activity of Lewis rats at 3, 6, 9, and 12 weeks after IN administration of 10^4^ FFU REVec-Luciferase. Dorsal and lateral images are shown at indicated time points. Total flux rate at each time point (photons/sec) is shown below. (n = 3). (**b**) *In vivo* luciferase activity of Lewis rats after IC administration of 10^4^ FFU REVec-Luciferase. Luciferase activity was measured as above at 2, 4, 6, 8, 10, and 12 weeks post-administration. Dorsal, ventral, and lateral images are shown at indicated time points. Total flux rate at each time point (photons/sec) is shown below (n = 3).

Since we did not observe tissue transduction by systemic administration of REVec in our preliminary experiment, we chose to inject the vector by intranasal (IN) and intracranial (IC) administration, which are most commonly used route of administration for the study of wild-type BoDV infection in rodent models^17,18^. Transmission competent REVec which encodes a firefly luciferase gene (REVec-Luciferase)^11^ was prepared by sonication of Vero cells persistently infected with REVec-Luciferase. Two-week-old Lewis rats were injected IN with Vero supernatant containing 10^4^ focus-forming unit (FFU) of REVec, and the vector distribution was analyzed using the *in vivo* imaging system (IVIS) at various time points post-administration (Fig. 1a). Onset of luciferase expression was at 6 weeks after injection, which increased in the brain, followed by spread into spinal cord at 9 weeks after injection. Decrease in luciferase expression was observed at 12 weeks post-administration.

In contrast, IC delivery of REVec to neonatal rats resulted in luciferase expression in the brain, spinal cord, and lower abdominal region at 2 weeks after injection, and similar expression was observed at 4, 6, 8, 10, and 12 weeks, with a gradual decline in total flux rate (Fig. 1b).

### Tissue tropism of transmission competent and defective vectors

**Figure 2:**
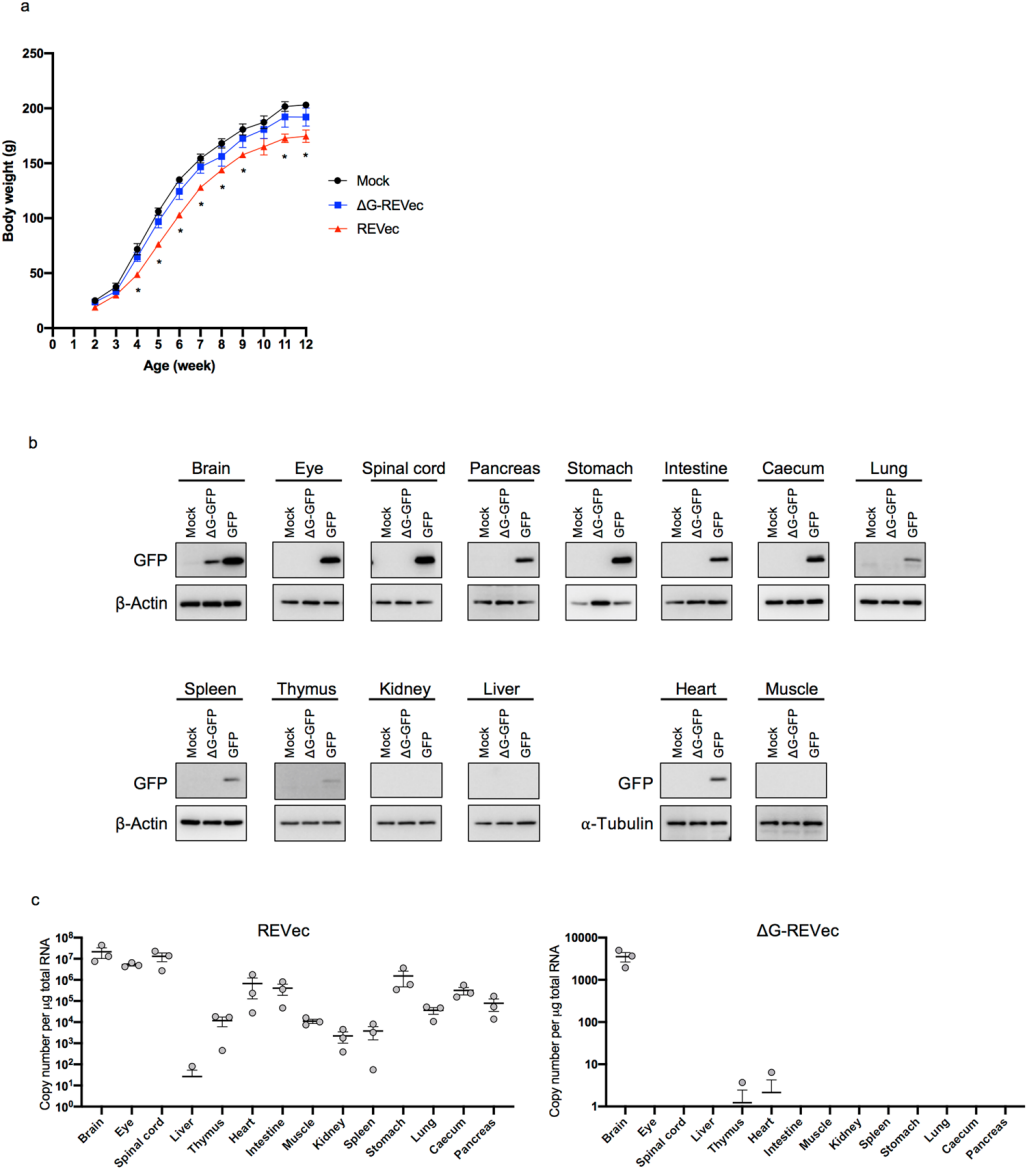
Tissue tropism of REVec and ΔG-REVec. (**a**) Body weight of Lewis rats administered IC with 10^4^ FFU REVec-GFP and ΔG-REVec-GFP (n = 3). (**b**) Animals inoculated with REVec-GFP or ΔG-REVec-GFP were sacrificed at 12 weeks after injection and the level of transgene expression was examined in indicated tissues by Western blot analysis for GFP, β-Actin, and α-Tubulin. Data are representative of triplicate experiment. Full-length immunoblots are presented in Supplementary Fig. S1. (**c**) Vector copy number per μg of total RNA in various tissues were determined by RT-qPCR. (n = 3).

We have previously developed a transmission defective vector lacking viral glycoprotein (G) gene (ΔG-REVec)^11^. As ΔG-REVec lacks viral envelope protein, it is capable of initially infecting cells and generating viral proteins, but it does not generate infectious progeny viral particles. Here, we conducted side-by-side comparisons of green fluorescent protein (GFP) encoding REVec and ΔG-REVec in their safety and efficiency of *in vivo* gene delivery. IC administration to neonatal rats was chosen as the delivery route since this route showed the most robust propagation of REVec *in vivo* (Fig. 1). While body weight of Lewis rats injected IC with ΔG-REVec was not statistically different from mock treated control animals over the course of 12 weeks, body weight of REVec administered animals was significantly lower than the control group, indicating minor pathogenicity of transmission competent REVec in these animals (Fig. 2a).

To compare long-term transgene expression from REVec and ΔG-REVec, different tissues were harvested at 12 weeks after administration and the GFP expression levels were analyzed by Western blot (Fig. 2b). REVec transduced the brain, eye, spinal cord, pancreas, stomach, intestine, and caecum, in addition to lower transgene expression in the lung, spleen, thymus, and heart. GFP expression was not observed in the kidney, liver, and muscle. In contrast, GFP expression was confirmed only in the brain of animals injected with ΔG-REVec. We further confirmed these results by quantifying the vector copy number by quantitative reverse transcription PCR (RT-qPCR) (Fig. 2c). The genome copy numbers of REVec exceeded 10^7^ copies in the brain and spinal cord. Over 10^6^ copy numbers were present in the eye and stomach; >10^5^ copy numbers in the heart, intestine, and caecum; >10^4^ copy numbers in the thymus, muscle, lung, and pancreas; >10^3^ copy numbers in the kidney and spleen. Lastly, >10^1^ copy numbers were detected in the liver in 1 out of 3 animals while it was below the detection level of qPCR in the rest of the animals. Similar to the results of Western blot analysis, the genome copy numbers of ΔG-REVec were generally much lower compared to those of REVec. We observed >10^3^ copy numbers of ΔG-REVec in the brain, while it was below the detection level of qPCR in other tissues examined (Fig. 2c).

### Transgene expression in different brain regions and cell types

**Figure 3:**
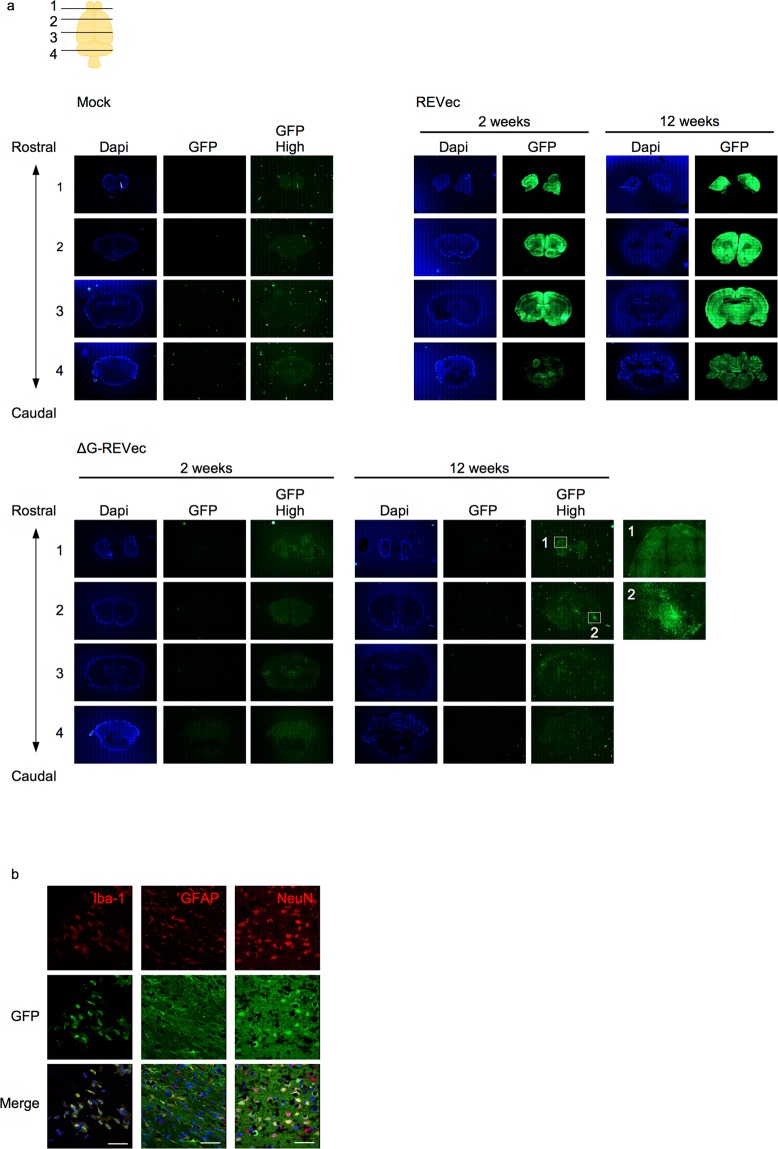
Transgene expression in different brain regions and cell types. (**a**) Immunohistochemical analysis of anterior to posterior coronal brain sections (20 μm) at 2 and 12 weeks post-injection with 10^4^ FFU REVec-GFP or ΔG-REVec-GFP. Higher exposure images of GFP (GFP High) are shown for mock and ΔG-REVec-GFP. Boxes 1 and 2 indicate high magnification images of selected brain regions showing magnified detail of GFP expression. (**b**) Immunohistochemistry of brain sections at 12 weeks after REVec injection co-labeled with GFP (green) and antibodies to microglia (Iba-1), astrocyte (GFAP), and neuron (NeuN). Cell specific markers are labeled in red and counterstained with DAPI. Scale bar, 50 μm.

Because the result of Western blot analysis and RT-qPCR indicated that both REVec and ΔG-REVec were capable of transducing brain, we next conducted histological analysis to determine whether they express GFP in any specific brain regions (Fig. 3a). To investigate the short- and long-term expression, brains of animals were harvested at 2 and 12 weeks after injection. Immunostaining of serial coronal brain sections revealed that REVec mediates transduction of different brain regions, including olfactory bulb, cerebral cortex, hippocampus, thalamus, hypothalamus, and cerebellum, with lower GFP expression in the hindbrain compared to other regions at both time points. On the other hand, GFP expression was not observed in the brain of ΔG-REVec administered animals at 2 weeks after administration. Weak GFP signal was detected in olfactory bulb and cortex region at 12 weeks post-administration with longer exposure time; however, the level of transduction by ΔG-REVec was significantly lower than that of REVec.

We next investigated different cell types that are susceptible to REVec by conducting co-immunostaining with antibodies to GFP and cell type specific markers for microglia ((Ionized calcium binding adaptor molecule 1 [Iba-1]), astrocytes (Glial fibrillary acidic protein ([GFAP]), and neurons (neuronal nuclei ([NeuN]) (Fig. 3b). GFP expression was confirmed in all three cell types examined, indicating a broad tropism of REVec in rodent brain.

### Analysis of immune cell infiltration in brain

**Figure 4:**
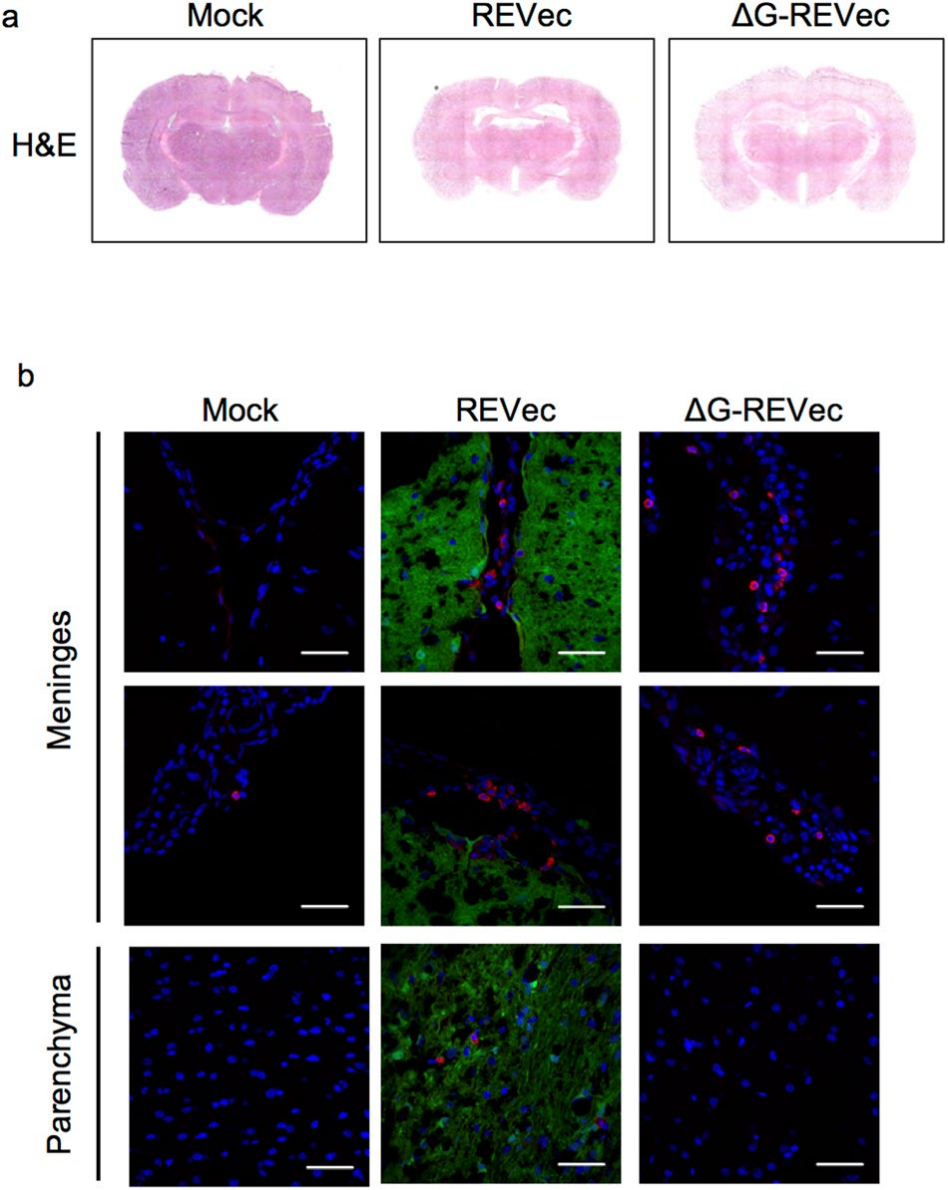
Histology analysis of brain at 12 weeks post-administration. (**a**) H&E staining of mock, REVec-GFP or ΔG-REVec-GFP brain sections (10 μm). (**b**) Immunohistochemistry of coronal brain sections (20 μm) stained with CD8 antibody (red) and counterstained with DAPI. CD8 T cells in brain parenchyma and meninges are shown. Scale bar, 50 μm.

It was reported previously that wild-type BoDV infection induces immune-mediated inflammation in brain^19^. To examine whether recombinant REVec and ΔG-REVec induce similar brain inflammation, we next conducted immunohistochemical analysis of cluster of differentiation (CD)8 T cells in brain at 12 weeks after administration. We did not observe any notable changes in haemotoxylin and eosin (H&E) staining of brain sections obtained from animals administered with REVec, or ΔG-REVec compared to mock treated animals (Fig. 4a). However, CD8 T cell infiltration was detected in the brain of both REVec and ΔG-REVec administered animals, but not in those that received mock treatment (Fig. 4b). Notably, CD8 T cells were mostly distributed in the meninges, with only a few cells penetrating the brain parenchyma in REVec administered animals. Although ΔG-REVec did not achieve efficient transduction of the brain, meningeal CD8 T cell infiltration was evident.

### Quantification of infectious vector titer in brain and cerebrospinal fluid

**Figure 5:**
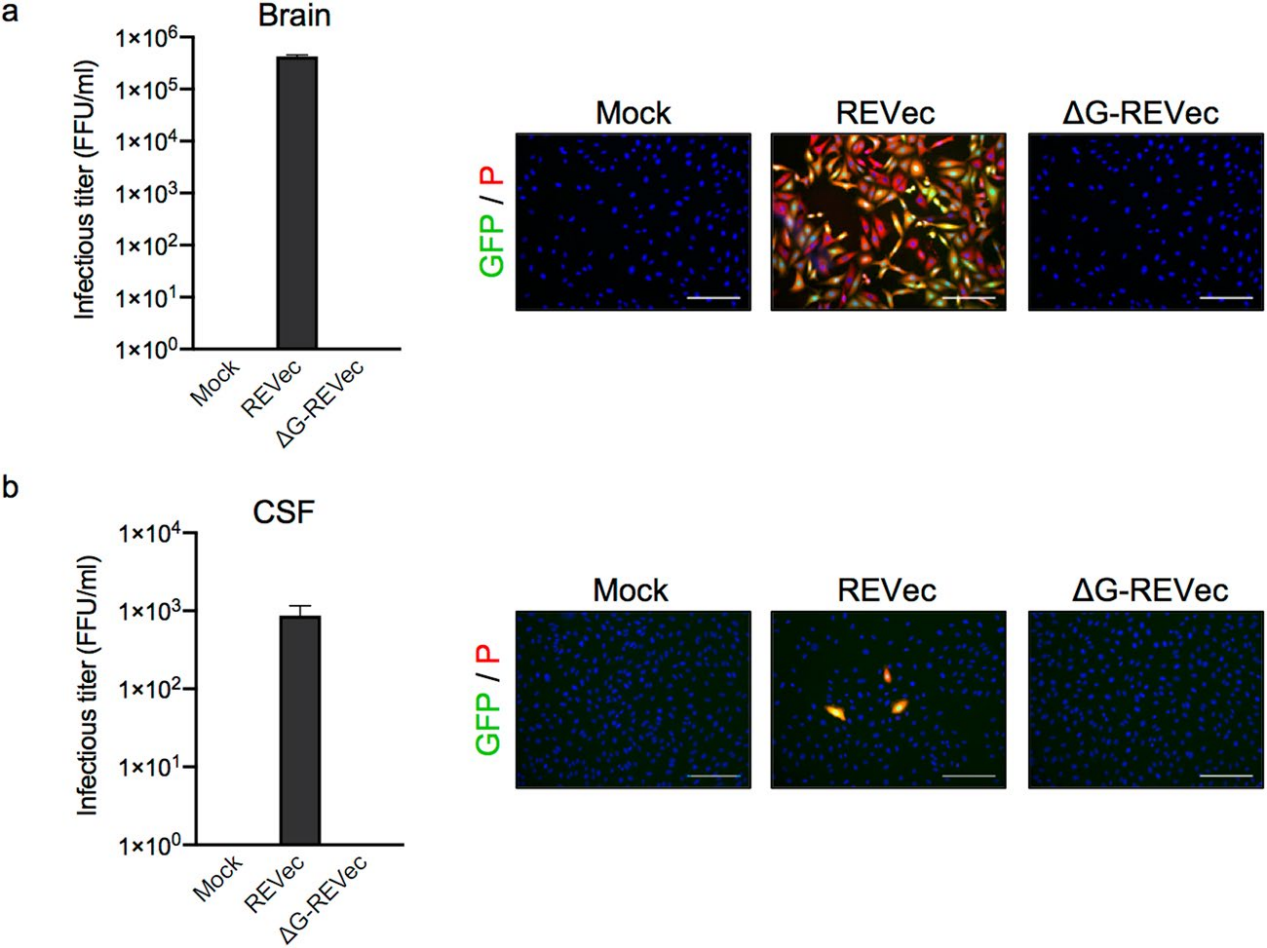
Quantification of infectious vector titer in brain and CSF. (**a**) Infectious vector titer in brain was quantified by focus-forming assay by inoculating Vero cells with serial dilutions of brain homogenates (n = 3). (**b**) Infectious titer of REVec in CSF was determined as above (n = 3). Representative images of Vero cells inoculated with brain homogenates or CSF and immunostained with BoDV-P antibody is shown to the right. Scale bar, 100 μm.

To assess transmission abilities of REVec and ΔG-REVec, infectious titer was determined in the brain homogenates and cerebrospinal fluid (CSF). More than 10^5^ FFU/ml of REVec was observed in the brain, while infectious vector was not detected in the brain after ΔG-REVec administration (Fig. 5a). Furthermore, infectious titer in the extracellular CSF was lower than that of the brain, with less than 10^3^ FFU/ml observed in animals administered with REVec, and infectious vector was absent in those administered with ΔG-REVec (Fig. 5b). As expected, infectious vector was not detected in the CSF after ΔG-REVec injection. Together, these results confirmed transmission competent and defective properties of 1^st^ and 2^nd^ generation REVec *in vivo*.

### Analysis of vector shedding

**Figure 6:**
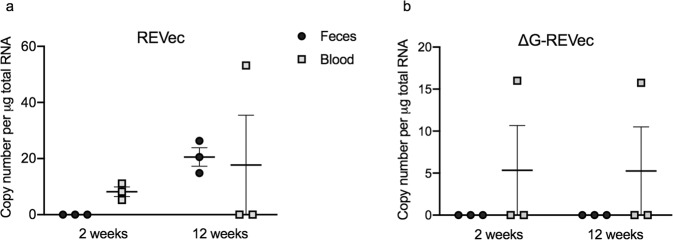
Vector shedding analysis. Vector copy number in feces and blood were determined by RT-qPCR at 2 and 12 weeks post-administration with (**a**) REVec-GFP or (**b**) ΔG-REVec-GFP (n = 3).

To assess vector shedding, fecal and blood samples were obtained from each animal and vector copy number was quantified by RT-qPCR. REVec genome in feces was below the detection level at 2 weeks, and less than 30 copies were detected at 12 weeks after injection (Fig. 6a). In contrast, less than 20 copies of REVec were present in blood at 2 weeks, and the vector genome was below the detection level in 2 out of 3 animals at 12 weeks post-administration.

In ΔG-REVec administered animals, vector genome was below the detection level of qPCR at both 2 and 12 weeks after administration in feces, and in 2 out of 3 animals in blood (Fig. 6b). One animal had less than 20 copies of vector genome in blood at both time points. Overall, we found either no or very low level of vector shedding in feces and blood after intracranial administration of REVec or ΔG-REVec.

### Analysis of anti-REVec antibodies in serum

**Figure 7:**
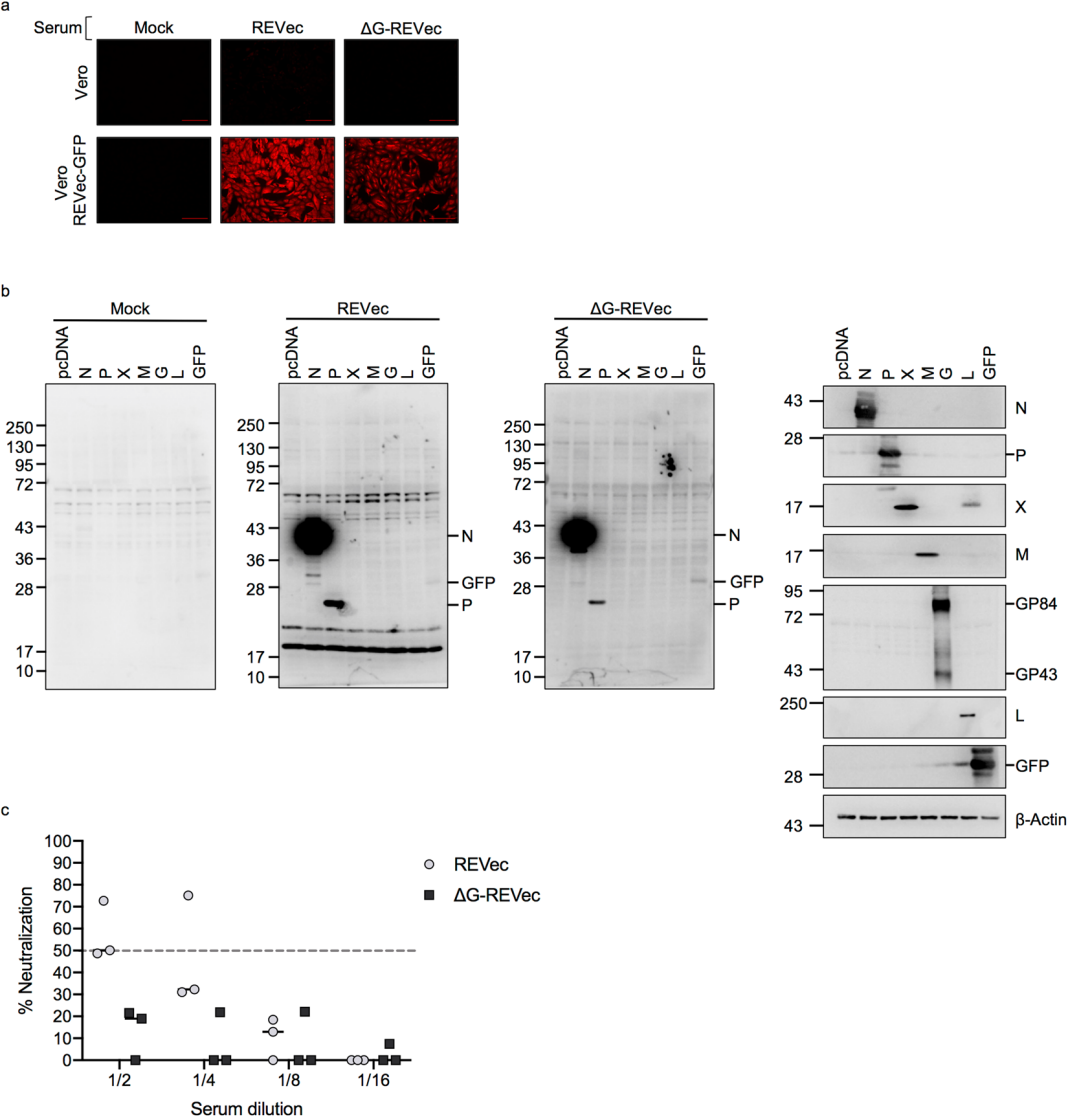
Detection of anti-REVec antibodies in serum. (**a**) Immunofluorescence analysis of Vero cells or Vero cells persistently infected with REVec-GFP using serum from mock, REVec, or ΔG-REVec inoculated animals. (**b**) Immunoblot analysis using serum from mock, REVec, or ΔG-REVec inoculated animals showing immunoreactivity of serum against each of the viral proteins and transgene. Western blot analysis using antibodies specific to the proteins of interest are shown to the right. Full-length immunoblots are presented in Supplementary Fig. S2. (**c**) REVec neutralization assay. The 100 FFU of REVec-GFP was pre-incubated with serial dilutions of serum obtained from mock treated, REVec, or ΔG-REVec injected animals, and inoculated on Vero cells. At 3 days post-inoculation, number of infected cells were quantified. Neutralization activity of serum obtained from REVec or ΔG-REVec were compared to control serum at each dilution. Data are presented as % neutralization compared to the control serum (n = 3).

To examine humoral immune responses against REVec or ΔG-REVec, we next tested for the presence of anti-REVec antibodies in serum at 12 weeks after administration. Serum obtained from REVec and ΔG-REVec injected animals both immunoreacted with Vero cells infected with REVec-GFP, while immunoreactivity was absent in uninfected Vero cells, indicating presence of anti-REVec antibodies in the serum (Fig. 7a). Next, immunoblot assay was conducted to further analyze immunoreactivity against each of the viral proteins. Serum from REVec or ΔG-REVec administered animals both detected nucleoprotein (N), P, and the transgene (GFP) (Fig. 7b). Antibodies against accessory protein (X), M, G, nor RNA-dependent RNA polymerase (L) protein were not detected by immunoblotting.

Lastly, we examined serum for neutralizing antibodies against REVec or ΔG-REVec. Serum was defined as having neutralizing activity if it showed more than 50% inhibitory activity compared to the control serum prepared from mock treated animals. Two out of three serums obtained from REVec administered animals exhibited >50% neutralization activity with 1/2 serum dilution compared to the control serum, indicating presence of low levels of neutralizing antibody. Neutralizing antibody was not detected in the serum of animals administered with ΔG-REVec (Fig. 7c).

## Discussion

Biodistribution analysis of viral vector system is essential for understanding its efficacy and safety. In particular, assessing *in vivo* activity of emerging RNA vector systems in preclinical animal models remain to be studied for approval of their clinical uses. We have previously reported transduction of rodent brains using BoDV-based REVec^11^, however, tissue tropism of the vector remained to be determined. In this study, we conducted for the first time detailed *in vivo* analysis of transmission competent REVec and transmission defective ΔG-REVec. In addition to gene transfer to the brain, we identified different tissues that are susceptible to REVec mediated transduction, including the eye, spinal cord, pancreas, stomach, intestine, caecum, lung, spleen, thymus and heart. High GFP protein levels were detected in the brain, eye, spinal cord, and stomach after REVec administration. These organs showed higher vector copy number (>10^6^) compared to other tissues examined. Although we did not detect GFP expression in liver and kidney, infectious virus has been detected in these organs in neonatal Lewis rats infected with wild-type BoDV^20^. Difference in infected tissues may be due to different sources of inoculated viruses. We used a cDNA-based BoDV strain for REVec generation, whereas brain-derived crude viruses were employed in previous studies. Furthermore, the genome of recombinant REVec contains some artificial mutations, including L116R and N1398D in the L gene (LRD mutations)^11^, which are known to enhance its polymerase activity in the mouse brains^21^, as well as an additional transcriptional cassette for GFP expression. We speculate that the difference in tissue tropism might be attributed to the changes in replication efficiency due to the sequence differences. The another possibility is difference in time after infection when the tissues were harvested for examination. In our experiment, tissues were harvested at 12 weeks post-injection, whereas previous study examined tissues at 30 weeks after infection. Therefore, longer incubation period may have allowed the virus to spread to other tissues that were not observed in this study.

*In vivo* imaging analysis of REVec showed the peak of luciferase activity at two weeks after IC injection, followed by decline over the course of 12 weeks. However, immunohistochemistry (IHC) analysis indicated otherwise, showing sustained level of GFP expression in different brain regions at both 2 and 12 weeks after injection. The discrepancy in these results could be explained by increase in bone density in growing animals, interfering with the emission of luminescence signal.

Investigation of gene transfer to different cell types in brain revealed that REVec mediates transduction of various cell types in the brain including neurons, astrocytes, and microglia. In experimental infection of wild-type BoDV in rodent models, various cell types, including astrocytes^18,22^, neurons^23^, and oligodendrocytes^24^ have been reported to be susceptible to infection. These results indicate that similar to the parental virus, REVec infects a broad range of cell types in brain.

Intracranial administration of REVec resulted in transduction of different organs distant from the brain. Previous studies on wild-type BoDV infection demonstrated spread of infection from brain to various organs via axonal and transneuronal transmission^23^. Therefore, we speculate that the vector propagated, in a similar manner, from brain to spinal cord then to peripheral nerves, eventually transmitting to various other organs. IN injection of REVec would have led to similar route of propagation after initial infection of olfactory nerves into the brain^17^.

We previously observed similar level of *in vitro* transduction by REVec and ΔG-REVec; however, efficiency of *in vivo* transduction was substantially different between these two vectors. Although we confirmed ΔG-REVec mediated GFP expression by Western blot analysis and confirmed presence of vector genome by qRT-PCR, our immunohistochemical analysis did not provide a strong evidence of efficient gene transfer to the brain by ΔG-REVec, with very low GFP expression observed in olfactory bulb and cerebral cortex. As GFP expression was low but was maintained for 12 weeks (Fig. 3a), low level of GFP expression by ΔG-REVec is most likely due to low efficiency of initial transduction rather than decrease in gene expression over time. In this study, we injected 10^4^ FFU of vector into each animal, which was the maximum dose that could be prepared by our current vector production protocol. In other viral vector systems, typical dosage of vector for *in vivo* gene delivery is much higher. For instance, more than 10^10^ vg of AAV vector is administered for effective *in vivo* gene delivery^25^. Further work will be required to improve the vector production system to increase the titer of ΔG-REVec, such as the use of cross-flow filtration system for vector concentration, and generation of cell lines that are capable of producing ΔG-REVec at a higher titer.

We found that neutralizing antibodies were present at very low level or absent in the animals at 12 weeks after administration. In wild-type BoDV infection, presence of neutralizing antibodies in infected animals has been controversial^23,26–29^. Neutralizing antibodies could be produced long time after infection^28,29^, which are directed against viral G and M proteins^29–31^. In current study, none of the serum analyzed showed immunoreactivity against G and M proteins, which may be the reasons for low neutralizing activities of serum in injected animals.

Although we did not observe behavioral alterations after IC administration of REVec and ΔG-REVec, which have been reported with experimental infection with wild-type BoDV^26^, significant decrease in body weight was observed in REVec injected animals. This indicates pathogenicity of recombinant REVec in rodent models at a dose tested, although the level of pathogenicity may be lower than the wild-type BoDV infection^26^. Notably, further IHC analysis indicated that injection of REVec resulted in CD8 T cell infiltration in the brain, which has also been reported for wild-type BoDV infection. Interestingly, CD8 T cell infiltration was similarly observed in the brain of animals administered with ΔG-REVec, which did not show any weight loss. Therefore, decrease in body weight caused by REVec is probably not due to the presence of these lymphocytes in the brain but caused by other side effects. On the other hand, the immune reaction in the brain after *in vivo* administration of both REVec and ΔG-REVec remains a significant concern, which must be addressed in future study. Studies with AAV vector have shown decrease in immunogenicity and increase in efficiency of transduction by increasing vector purity^32–34^. Similarly, the immunogenicity of REVec and ΔG-REVec could be caused by the impurities present in vector preparation. The traditional method of REVec preparation involves sonication of vector producing cells, resulting in contamination of a large amount of cell-derived impurities and vector-derived viral proteins such as free N and P proteins in final vector preparation. Therefore, increasing vector purity may be essential to decrease immunogenicity and to increase the potency of REVec.

In summary, our findings demonstrate, for the first time, difference in tissue tropism and efficiency of *in vivo* gene delivery by the first generation REVec and second generation ΔG-REVec, and challenges that need to be overcome in the future study. These findings have important implications for the use of REVec for *in vivo* gene therapy. Since REVec was capable of achieving long-term transduction of different cell types in the brain, generation of attenuated vector to reduce vector-associated pathogenesis and improvement in the efficiency of gene transfer would allow for successful applications of ΔG-REVec for treatment of CNS diseases.

## Methods

### Cell culture

Vero, Vero cells stably expressing BoDV G (Vero-BG), and Vero REVec-GFP cells were cultured in low glucose Dulbecco’s modified Eagle’s medium (DMEM) (Nacalai Tesque, Kyoto, Japan) supplemented with 2% fetal calf serum (FCS). 293 T cells were cultured in high glucose DMEM (Thermo Fisher Scientific, Waltham, MA, USA) supplemented with 10% FCS.

### Generation of recombinant REVec

REVec-Luciferase, REVec-GFP, and ΔG-REVec-GFP were generated as previously described^11^. Briefly, transmission competent REVec was generated by co-transfection of 293 T cells with pFct-BoDV P/M-Luciferase or pFct-BoDV P/M-EGFP vector genome plasmid and helper plasmids (N, P, L) using Lipofectamine 2000 (Thermo Fisher Scientific), and overlaid with puromycin resistant Vero cells. At 5 days post co-culture, cells were treated with puromycin and passaged until majority of the cells became positive for vector production. ΔG-REVec was generated by co-transfection of 293 T cells with pFct-BoDV ΔGLLP/M-GFP vector genome with helper plasmids and G plasmid followed by co-culture with puromycin resistant Vero-BG cells.

### Western blot

To prepare protein samples from tissues, frozen tissues were immediately homogenized in radioimmunoprecipitation assay (RIPA) buffer supplemented with cOmplete protease inhibitor cocktail (Roche, Basel, Switzerland) using BioMasherII (Nippi. Tokyo, Japan). After cell lysis on ice for 1 hr, tissue homogenates were centrifuged at 14,000 g for 15 min, and the supernatant was collected. Protein concentration was determined using Pierce BCA protein assay kit (Thermo Fisher Scientific), and 10 μg of each protein was separated on Sodium dodecyl sulfate polyacrylamide gel electrophoresis (SDS-PAGE), and transferred to a nitrocellulose membrane. Membranes were blocked with blocking one reagent (Nacalai Tesque), followed by overnight incubation with following primary antibodies. GFP-living colors A.v. monoclonal antibody JL-8 (Clontech Laboratories, Mountain View, CA, USA), monoclonal anti-β-Actin antibody clone AC-15 (Sigma, St. Louis, MO, USA), and monoclonal anti-α-Tubulin antibody clone B-5-1-2 (Sigma). Membranes were washed with TBST (Tris-buffered saline, 0.1% Tween 20) and incubated with the following secondary antibodies from Jackson ImmunoResearch (West Grove, PA, USA): Peroxidase affinipure donkey anti-mouse IgG, and peroxidase affinipure donkey anti-rabbit IgG. After 1 hr of secondary antibody incubation, membranes were washed with TBST and developed using enhanced chemiluminescence (ECL) prime Western blotting detection reagent (GE Healthcare, Pittsburgh, PA, USA).

### Determination of infectious titer

Infectious titer was determined by focus-forming assay by incubating Vero cells with serial dilutions of brain homogenates or CSF. At 72 h post-infection, cells were fixed with 4% paraformaldehyde, permealized in 0.4% Triton-X, and blocked with phosphate buffered saline (PBS) containing 2% bovine serum albumin (BSA), followed by incubation with primary antibody against BoDV-P. After 1 hr of incubation with primary antibody, cells were washed with PBS, and incubated with goat anti-rabbit IgG conjugated with Alexa Fluor 555 (Thermo Fisher Scientific) for 1 hr. Cells were washed with PBS, and counterstained with 4’,6’-diamidino-2-phenylindole (DAPI) (Thermo Fisher Scientific). Fluorescence images were taken with Eclipse TE2000-U inverted microscope (Nikon, Shinagawa, Japan), or with a Ti-E inverted microscope with a C1 confocal laser scanning system (Nikon).

### Animal experiment

All experiments involving animals were approved by the institutional animal care and use committee at Kyoto University. All experiments were performed in accordance with relevant guidelines and regulations at Kyoto University. Pregnant LEW/SsNSlc rats were obtained from SLC Japan (Shizuoka, Japan). For IC administration, vector was administered to neonatal Lewis rats within 24 hr after birth. Prior to injection, each neonate underwent hypothermic anesthesia by previously described protocol^35^. Once hypothermic anesthesia was induced, 10^4^ FFU of REVec or ΔG-REVec were administered using Hamilton syringe (Hamilton Company, Reno, NV, USA) with 27 G×1/2SB needle (TOP, Tokyo, Japan). Supernatant prepared by sonication of uninfected Vero cells was used for mock treatment. Immediately after the procedure, neonates were placed on a warming pad for a recovery. For IN administration, 2 week-old female rats were anesthetized with 3% isoflurane and oxygen, and administered with 10^4^ FFU of REVec or ΔG-REVec using Gilson P-20 pipette.

### *In vivo* bioluminescence imaging

Rats were anesthetized with 3% isoflurane and oxygen prior to intraperitoneal (IP) injection with the VivoGlo Luciferin (Promega, Madison, WI, USA) at a dose of 150 μg/g of body weight. Approximately 5 min after injection of the substrate, each rat underwent *in vivo* imaging using IVIS Lumina III (PerkinElmer, Waltham, MA, USA). Images were captured from different positions (dorsal, ventral, lateral) and analyzed using the living image software. Luminescence of dorsal images were quantified by designating a region of interest at each time point and determining the total flux of luminescence as described previously^36^. Serial images were acquired at 3, 6, 9, and 12 weeks after IN administration, and at 2, 4, 6, 8, 9, 10, and 12 weeks after IC administration.

### Histology and immunohistochemistry

To prepare tissues for IHC, animals were anaesthetized and intracardially perfused with PBS solution followed by 4% paraformaldehyde (PFA) PBS (FUJIFILM Wako Pure Chemical Corporation, Osaka, Japan). Brains were further immersion fixed in 4% PFA PBS solution for 48 hr, cryoprotected in 10–30% sucrose PBS solution, and embedded in Tissue-Tek OCT compound (Sakura Finetek, Tokyo, Japan). Coronal brain sections (10–20 μm) were prepared using Leica cryostat, and mounted on MAS-GP glass slides (Matsunami Glass, Osaka, Japan). Sections were immersed in PBS for 5 minutes, permeabilized in 0.1% TritonX-100 in PBS (0.1% PBS-T) for 30 min, and blocked with Blocking One Histo (Nacalai Tesque) for 10 min. Following primary antibodies were used for IHC: Anti-GFP pAb (MBL, Nagoya, Japan), goat polyclonal Iba1 antibody (Novus Biologicals, Centennial, CO, USA), GFAP mouse mAb (Cell Signaling Technology, Danvers, MA, USA), Anti-NeuN antibody clone A60 (EMD Millipore, Temecula, CA, USA), and Anti-CD8a mouse IgG (BioLegend, San Diego, CA, USA). Tissue sections were incubated in primary antibodies diluted in 1% BSA/0.1% PBS-T overnight, washed with 0.1% PBS-T, followed by incubation in following secondary antibodies. Goat anti-mouse IgG conjugated with Alexa Fluor 555; goat anti-rabbit IgG conjugated with Alexa Fluor 488; donkey anti-goat IgG conjugated with Alexa Fluor 555 (all from Thermo Fisher Scientific). After 1–2 hr of incubation in secondary antibodies, tissues were washed with 0.1% PBS-T, and mounted in VECTASHIELD hardset antifade mounting medium with DAPI (Vector Laboratories, Burlingame, CA, USA). GFP and DAPI images of entire brain sections were acquired using EVOS FL auto imaging system (Thermo Fisher Scientific). Double-immunostained sections were analyzed by confocal microscopy. H&E staining was conducted using H&E stain kit (ScyTek Laboratories, West Logan, UT, USA) as per manufacturer’s instructions.

### Serum analysis and neutralization assay

For serum analysis, protein samples were prepared from 293 T cells transfected with empty plasmid, individual viral antigens (N, P, X, M, G, L), or transgene (GFP). Proteins were separated on SDS-PAGE, transferred to nitrocellulose membranes, and incubated with mock, ΔG-REVec or REVec serum overnight, followed by incubation with goat anti-Rat IgG-horseradish peroxidase (HRP) secondary antibody (Thermo Fisher Scientific). As a positive control, Western blot analysis was conducted using antibodies against specific proteins of interest (N, P, M, G, GFP, and β-Actin). FLAG-tagged X and L genes were transfected and protein expression detected using FLAG antibody due to lack of available antibodies.

For neutralization assay, serum collected from each animal was incubated at 56 °C for 30 min for inactivation of complement. 100 FFU of REVec-GFP was pre-incubated with serial two-fold dilutions of heat inactivated serum for 1 hr at room temperature, and then inoculated on Vero cells. Following 2 hr incubation, cells were replaced with fresh medium and further cultured for 3 days. The percentage of neutralization activity was determined by quantifying the number of GFP positive cells using Tali image cytometer (Thermo Fisher Scientific). The serum which reduced the number of GFP positive cells by >50% compared to control (mock) serum was defined as having neutralization activity.

### Determination of vector copy number by qRT-PCR

Tissue samples were collected from each animal and immediately stored in RNALater stabilization solution (Ambion, Austin, TX, USA). RNA was isolated from all tissues except for the heart and skeletal muscle by homogenization in 1 mL of TRIzol reagent, followed by centrifugation for 5 min at 12,000 g. The resulting supernatant was mixed with chloroform, incubated at room temperature for 3 min, next centrifuged for 15 min at 12,000 g. The resulting aqueous phase was mixed with 70% ethanol and the total RNA was isolated using RNeasy mini kit (QIAGEN, Hilden, Germany) according to manufacturer’s instructions. RNA was isolated from the heart and skeletal muscle using RNeasy fibrous tissue mini kit (QIAGEN). RNA was isolated from whole blood using TRIzol LS reagent. Fecal samples were first homogenized in PBS using Biomasher V (Nippi), followed by RNA isolation using TRIzol LS reagent.

For determination of vector copy number, BoDV genome template was prepared by *in vitro* transcription using MEGAscript T7 transcription kit (Thermo Fisher Scientific) and following primer set: BoDV T7-vRNA synthesisF (GTTGCGTTAACAACAAACCAATCAT); BoDV T7-vRNA systhesisR (+T7p) (TAATACGACTCACTATAGGGGCAACATGGGTGCAGAGGTCCCAA). cDNA was prepared using one microgram of total RNA using SuperScript III (Thermo Fisher Scientific) and following RT primer: BoDV-strand gRNA RT (GGCCGTCATGGTGGCGAATGTTGCGTTAACAACAAACCAATCAT).

Quantitative PCR analysis was performed using Power SYBR Green PCR master mix (Applied Biosystems, Waltham, MA) and analyzed on the StepOnePlus real-time PCR system (Applied Biosystems). The cycling conditions were as per manufacturer’s instructions: 95 °C for 10 min followed by 40 cycles of 95 °C for 15 sec and 60 °C for 1 min. Following primers were used for qPCR: BoDV-strand qPCR F (GGCCGTCATGGTGGCGAAT); BoDV-strand qPCR R (AATCTTGGTCCTCCATGGCATC). Vector copy number was determined by generating a standard curve using cDNA standards prepared from *in vitro* transcribed RNA.

## Supplementary information

Supplementary Information.
