# Supplementary material for: In vivo biodistribution analysis of transmission competent and defective RNA virus-based episomal vector
Source: Sci Rep. 2020 Apr 3;10:5890. doi: 10.1038/s41598-020-62630-7 (PMC7125079; doi:10.1038/s41598-020-62630-7)
Supplement: Supplementary file 1 — Supplementary Information. [file 41598_2020_62630_MOESM1_ESM.pdf]

Supplementary information

In vivo biodistribution analysis of transmission competent and defective RNA virus-based episomal vector

Yumiko Komatsu, Chiaki Tanaka, Ryo Komorizono, and Keizo Tomonaga

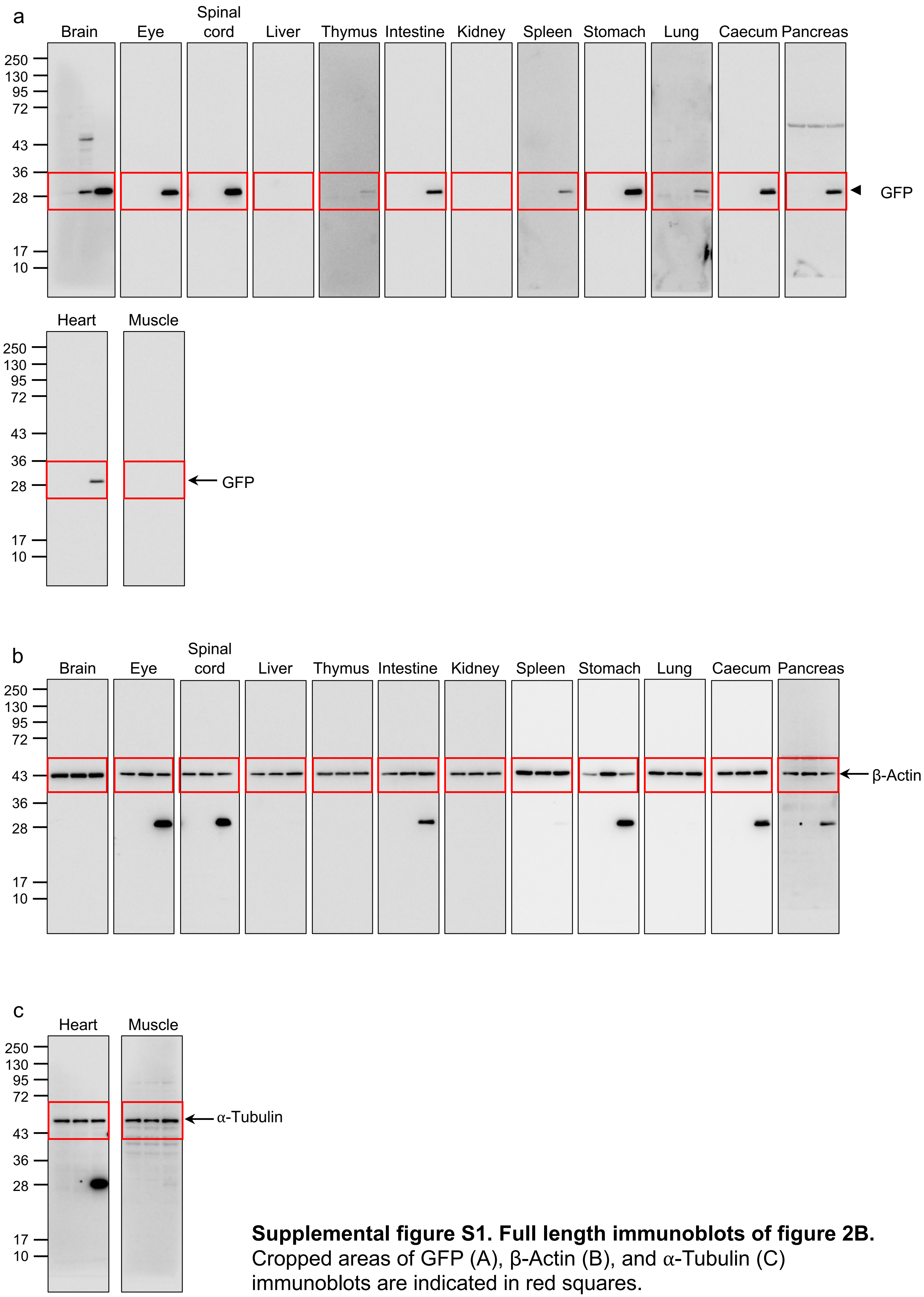

Supplemental figure S1. Full length immunoblots of figure 2B. Cropped areas of GFP (A), β-Actin (B), and α-Tubulin (C) immunoblots are indicated in red squares.

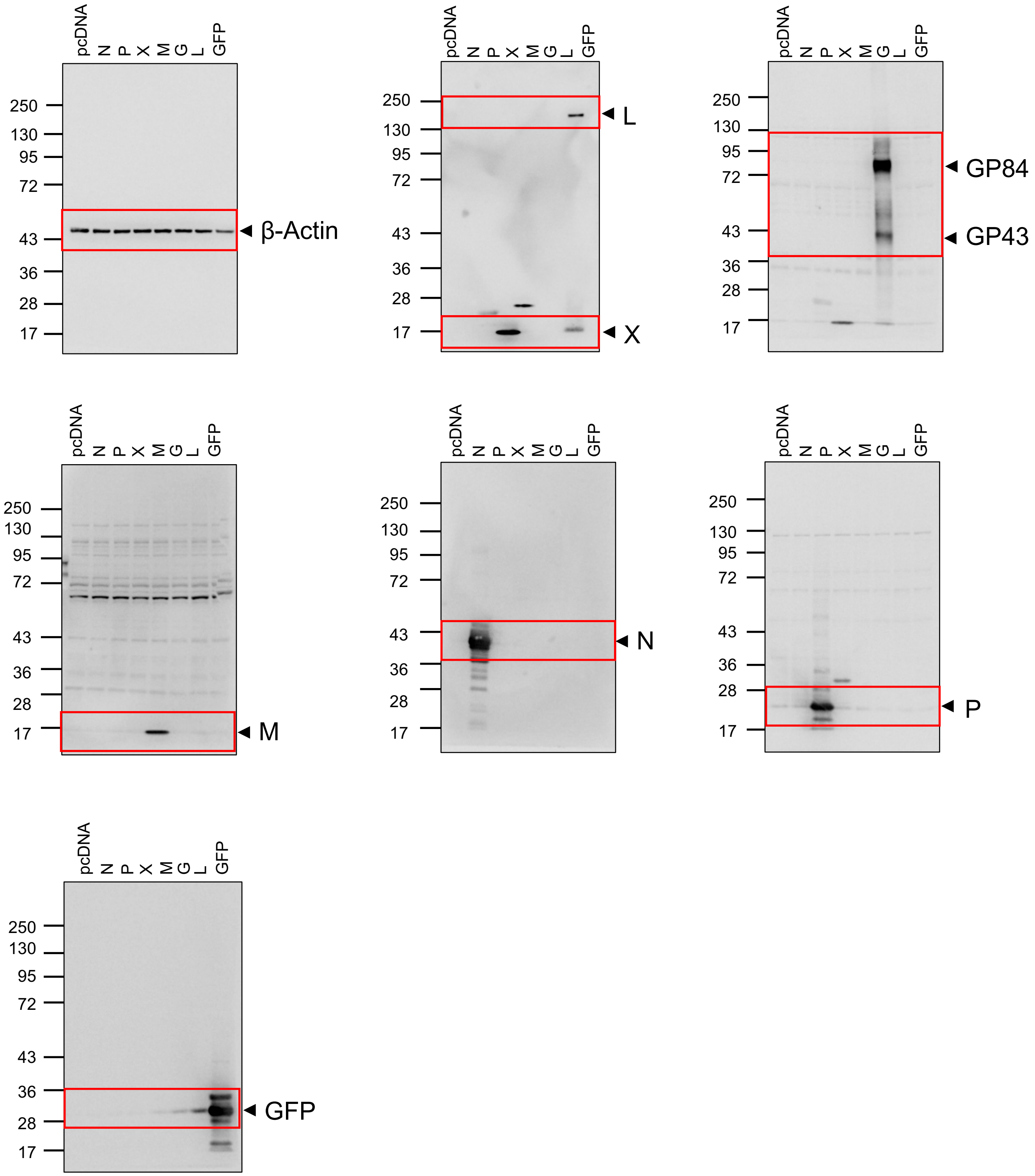

Supplemental figure S2. Full length immunoblots of figure 7B.
Cropped areas of β-Actin, X, GP84, GP43, M, N, P, and GFP immunoblots are indicated in red squares.
